# Exploring the general acceptance factor for shared automated vehicles: the impact of personality traits and experimentally altered information

**DOI:** 10.3389/fpsyg.2025.1531386

**Published:** 2025-04-09

**Authors:** Ole Aasvik, Pål Ulleberg, Marjan Hagenzieker

**Affiliations:** ^1^Institute of Transport Economics, Oslo, Norway; ^2^Department of Psychology, University of Oslo, Oslo, Norway; ^3^TU Delft, Department of Transport and Planning, Delft, Netherlands

**Keywords:** autonomous vehicles, acceptance, experiment, personality, survey

## Abstract

**Introduction:**

Shared automated vehicles (SAVs) could significantly enhance public transport by addressing urban mobility challenges. However, public acceptance of SAVs remains under-studied, particularly regarding how informational factors and individual personality traits influence acceptance.

**Methods:**

This study explores SAV acceptance using data from an experimental survey of 1902 respondents across Norway. Participants were randomly presented with different informational conditions about SAV services, manipulating vehicle autonomy (fully autonomous vs. steward onboard), seating orientation (facing direction of travel vs. facing other passengers), and ethnicity of co-passengers. Personality traits from the Five Factor Model (FFM) and Social Dominance Orientation (SDO) were assessed. The General Acceptance Factor (GAF), derived from the Multi-Level Model of Automated Vehicle Acceptance (MAVA), was used as the primary outcome measure.

**Results:**

No significant main or interaction effects were found from the experimentally altered information conditions. However, personality traits significantly influenced acceptance. Specifically, higher openness and agreeableness positively predicted SAV acceptance, while higher neuroticism and social dominance orientation negatively predicted acceptance.

**Discussion:**

The absence of experimental effects suggests either a limited role of the manipulated factors or insufficiently robust manipulations. Conversely, the substantial impact of personality traits highlights the importance of psychological factors, particularly trust, openness, and social attitudes, in shaping SAV acceptance. These findings emphasize the need for tailored communication strategies to enhance SAV uptake, addressing specific psychological profiles and fostering trust in automation.

## 1 Introduction

The recent surge in automated^1^ vehicle (AV) technology offers more than just a leap in engineering; it has the potential to reshape how modern societies organize transportation. In ever more urbanized societies, many cities face great challenges to public road infrastructure. By optimizing capacity utilization and reducing congestion, shared AVs (SAVs) could deliver great societal benefits for these kinds of issues (Iclodean et al., 2020; Silva et al., 2022). Promoting ride sharing in an efficient, less accident-prone, green transport system may hold a key to several modern challenges. However, technical prowess alone cannot guarantee success. Psychological factors play an equally critical role in shaping whether people are willing to adopt this technology. However, only one in 10 studies of SAV acceptance include socio-psychological factors (Azad et al., 2019; Cohen et al., 2020; Greifenstein, 2024). The real implementation of SAV systems is still in its’ infancy, and we meanwhile need to improve the understanding of SAV acceptance to harvest the full potential benefits. This study addresses that need by examining how subtle changes in information about a future SAV service may influence public perceptions, and by testing whether personality traits further shape these responses.

## 2 Acceptance of automated vehicles

The recent growth in research on AV acceptance has been sparked by technological advancements. There have been many SAV pilots being conducted in Europe (Hagenzieker et al., 2021). Most of these vehicles are small, with few passengers, and driving slower than 21 km/h. Additionally, most of them have only been operating at SAE level 3, meaning “conditionally automated” driving (SAE International, 2021). Because of these limitations, and their limited deployment, research on SAV acceptability is greatly dependent on the knowledge of participants. Researchers may also inform research participants, and this info may have its’ own bias (Delbosc, 2022). Recently, driverless taxi services have been deployed with California, USA and Wuhan, China as the most prominent examples and with an increasing user base. These early services have, however, not been without public outcry, and many call for informational campaigns about the safety and reliability of the vehicles (Jin and Roy, 2024; Shepardson, 2024; Zhanhang and Jingyang, 2024). Investigating how different informational messages impact the perceptions of SAV services may be critical to improve SAV acceptance.

Studies have found individuals to be largely optimistic about the future of AVs. This optimism increases as they engage in reflective thought about AVs and experience them in real-world settings (Liljamo et al., 2018; Nordhoff et al., 2022; Schoettle and Sivak, 2014; Tennant et al., 2016). However, an algorithm aversion has been documented, where people disfavor automated services over human services, and not all pilot testing is suited to grow optimism (Aasvik et al., 2024b; Shariff et al., 2021). Different kinds of AVs are perceived differently, where people tend to prefer AVs to take the form of shuttles over buses or robotaxis (Chng et al., 2022; Salladarré et al., 2021). Seating arrangements within SAVs may impact perceptions so that rear-facing seats negatively impact trust and expected comfort (Nordhoff et al., 2019a; Paddeu et al., 2020). This could be explained by this configuration implying less control over the driving style of the SAV (Diels et al., 2016). However, these results are inconsistent and need further investigation (Paddeu et al., 2021). Having a steward onboard the vehicles may decrease their popularity (Winter et al., 2019), while others suggest that this effect is only true for some population segments (Kyriakidis et al., 2020). Furthermore, the social situation inside small AVs may be a deterrent for some groups, i.e., men or women with children, that report higher levels of discriminatory attitudes towards co-riders (Middleton and Zhao, 2019; Sanguinetti et al., 2019). Different technologies and contexts will generate different results and researchers should be careful with the way they present the SAVs, for example regarding the interior design or autonomous capabilities of SAVs. Little is known about perceptions of SAVs and their characteristics, and experimental studies should be conducted to illuminate this issue further.

The Unified Theory of Acceptance and Use of Technology (UTAUT) has been a widely successful framework for explaining and predicting technology acceptance over the last decades (Venkatesh et al., 2016). This framework has been adapted to the realm of AV acceptance (Bellet and Banet, 2023; Madigan et al., 2017; Nordhoff et al., 2019b). The Multi-Level Model of Automated Vehicle Acceptance (MAVA), is informed by 124 previous studies on AV acceptance (Nordhoff et al., 2019b). It provides an exhaustive framework for explaining AV acceptance. However, the model is vast, and little is known about how well research about AVs in general fit the context of shared vehicles introduced in the public transport system. There are also unanswered questions about the novel social situations that arise in small, shared vehicles (Greifenstein, 2024; Sovacool and Axsen, 2018). Additionally, the prospect of mobility as a service, where vehicles pick you up wherever instead of traditional bus stops, can further exacerbate the intimate feeling of such vehicles.

The MAVA and UTAUT models both predict actual use of technology through intention to use (Nordhoff et al., 2019b; Venkatesh et al., 2016). In the MAVA, intention is predicted by a set of nine meso-level predictors. These are performance expectancy, effort expectancy, facilitating conditions, safety, service and vehicle characteristics, social influence, hedonic motivation, perceived benefits, and perceived risks. Additionally, individual difference factors are suggested as moderators, such as socio-demographics, travel behavior, and personality. Some research suggest that acceptance of AVs is adequately explained by a single General Acceptance Factor (GAF), as the UTAUT elements often share large correlations (Blut et al., 2022; de Winter and Nordhoff, 2022; Nordhoff et al., 2018). Validation attempts suggests that the single GAF is the best statistical representation of the many variables from the UTAUT and MAVA frameworks (de Winter and Nordhoff, 2022). Similar results have recently been found in a Norwegian survey (Aasvik et al., 2024c). Like other psychological research, such as within the general intelligence factor *G*, there seems to be some construct proliferation where there is a multitude of constructs for similar phenomena (de Winter and Nordhoff, 2022; Schmidt, 2017). Issues regarding sibling constructs, jingle-jangle-fallacies and theory building may exacerbate the crisis of replication and reputation in the field (de Winter and Nordhoff, 2022; Fried, 2020; Lawson and Robins, 2021; Smaldino, 2017). Not all research supports the notion of a GAF, and there is need for further research to clarify how best to conceptualize these factors (Kacperski et al., 2021; Rahimi et al., 2020).

The last 5 years have seen a great increase in publication about AV acceptance. Although much this has been without clear theoretical grounding, there are numerous publications addressing factors from established theories like Technology Acceptance Model, Theory of Planned Behavior, or the UTAUT (Greifenstein, 2024). Trust in the technology and perceived safety have consistently emerged as key drivers of acceptance (Aasvik et al., 2024a; Aasvik et al., 2024c; Choi and Ji, 2015; Zhang et al., 2020; Zhang et al., 2021). Similarly, perceived usefulness of AVs, the degree to which people expect AVs to be beneficial, convenient, or efficient, is strongly associated with positive attitudes and intentions to use, aligning with traditional technology acceptance frameworks (Aasvik et al., 2024c; Gopinath and Narayanamurthy, 2022; Li et al., 2024; Wiefel, 2020). Perceived risk is sometimes used as a separate, important construct including factors like data security or hacking, ethical issues regarding accidents, and concerns about disease following the COVID/19 pandemic (Gkartzonikas and Gkritza, 2019; Othman, 2021; Zhang et al., 2021). Some research finds that young people and men tend to be more optimistic, but that sociodemographic information is far less important than psychosocial factors (Becker and Axhausen, 2017; Nordhoff et al., 2019b; Nordhoff et al., 2022). Several of the most important predictors of AV acceptance correlate significantly, lending further credence to the idea of a general acceptance factor (GAF). The field of technology acceptance research has been called chaotic, due to the many frameworks and factors investigated, and the lack of proper contextualization in an emerging research field (Bagozzi, 2007; Blut et al., 2022). The field is in need for consolidation and standardization across contexts. More research into the GAF and how to best represent it could therefore be a fruitful way forward. Within this broader landscape, shared automated vehicle (SAV) research is a smaller, nascent subfield. Many of the same factors will be of importance, but there is reason to believe that the understudied social situation may be governed, at least partly by some other factors. In the following, we will present to theories we believe may help connect the AV acceptance research with established psychological research.

### 2.1 The five factor model of personality (FFM) and SAV acceptance

In studies on acceptance of AVs, the personality-related factors included have mostly been trust, technological savviness, locus of control, sensation seeking, and similar factors (Nordhoff et al., 2019b). The MAVA also suggests these personality factors as moderating variables influencing the intention to use AVs. Often these personality-related factors are reported as significant, particularly trust and technological savviness or personal innovativeness (Blut et al., 2022; Nordhoff et al., 2019b; Zhang et al., 2020). There is, however, little research investigating the most influential theory of personality in psychology, the FFM of personality in this context (Larsen and Buss, 2010). The FFM sorts respondents using five bi-dimensional traits: extraversion, neuroticism, openness, conscientiousness, and agreeableness. The current study aims to address this contrast between the most popular personality traits tested in AV acceptance and the most popular theory in psychology. The goal is to offer this tested theory as a standardized measure of personality in AV acceptance research, instead of relying on a multitude of separate, disorganized factors.

These traits are thought to be stable descriptors of how people generally behave over time. Some previous research has suggested that the FFM play little of a role for people’s opinions of and intentions to use AVs (Kyriakidis et al., 2015; Payre et al., 2014). There are several reasons why this is not necessarily applicable to the current study. First, the models they tested did not include any of the other factors from the MAVA, nor its’ interaction with SAVs’ characteristics. Second, the dependent variable was opinion of automated driving, and not intention to use SAVs.

Third, other research has found some effects of the FFM personality traits (McElroy et al., 2007). People who score higher on neuroticism had more safety concerns, find SAVs less useful, and are less likely to want to use SAVs (Barnett et al., 2015; Devaraj et al., 2008; Payre et al., 2014; Svendsen et al., 2013). Indeed, neuroticism has been shown to predict lower intentions to use AVs and negative feelings about robots in general (Charness et al., 2018; Khan et al., 2014). It seems that trait neuroticism may enhance the need for trust in evaluating SAV services.

Scoring higher on openness and extraversion may boost self-driving car acceptance (Charness et al., 2018; Qu et al., 2021). Other research suggest no such effect (Barnett et al., 2015). Yet other investigations of AVs did find that those who score higher in sensation seeking and openness were more likely to trust AVs and had a higher intention to use, while others do not find that (Barnett et al., 2015; Bellet and Banet, 2023; Devaraj et al., 2008; Payre et al., 2014; Svendsen et al., 2013; Zhang et al., 2020). Sensation-seeking is typically placed under extraversion or openness and may typify those who are oriented towards the external world and seek novel experiences (McCrae and Costa, 2003; Roberti, 2004). Agreeableness and conscientiousness have been found to correlate positively with trust in general automation (Chien et al., 2016), and conscientiousness may predict increased concern about the disruptive nature of AV technology and lower intention to use (Charness et al., 2018). Those high in conscientiousness may worry about reliability, efficiency, and ease of use (Barnett et al., 2015; Devaraj et al., 2008; Qu et al., 2021). Propensity to trust is often organized under agreeableness in FFM, and interpersonal trust may be similar to a person’s trust in automation for advanced systems (McCrae and Costa, 2003; Sheridan, 2019). Agreeableness may thus be particularly important for automated systems. Agreeableness is strongly correlated with interpersonal trust, which may improve ridesharing attitudes (Graziano et al., 1996). The social situation arising in SAVs may exacerbate these effects, and perhaps also engage other factors such as agreeableness and other facets of extraversion. This research is largely done outside the context of AVs and completely outside the MAVA framework. More research is needed to investigate which of these claimed relationships are applicable to the Norwegian context of SAV use.

### 2.2 Ridesharing and the potential role of social dominance theory (SDT)

An individual’s social dominance orientation (SDO) reflects their intergroup attitudes. SDO is a leading individual difference factor from social psychology that seeks to predict and explain how groups of people interact in society (Kleppestø et al., 2020; Pratto et al., 1994). The Social Dominance Theory (SDT) posits that humans tend to organize into group-based social hierarchies with some groups having greater power than others. SDO measures the degree to which an individual supports or opposes these hierarchies. This measure has been found similar, but separate from right-wing authoritarianism and factors from the Big Five personality inventory (Duckitt, 2006; Ekehammar et al., 2004). SDO predicts many different manifestations of prejudicial attitudes that enhance or strengthen group based social hierarchy (Kleppestø et al., 2020). This also impacts perceptions about political and economic ideology, more cultural elitism, more racism, more sexism, more chauvinism, less support of gay/lesbian rights, less empathy, less altruism, and a generally lower concern for others (Bizer et al., 2012; Duckitt, 2001; Kunst et al., 2014; Pratto et al., 2013; Sibley et al., 2007; Sidanius et al., 1994; Sidanius et al., 1994). Research suggests that individuals who score high on SDO want more social distance from people they perceive to be lower in the hierarchy, such as people with mental illness (Bizer et al., 2012; Crisp et al., 2000; Johansson and Kunst, 2017; Kvaale and Haslam, 2016; Sidanius et al., 1994). Therefore, we want to explore whether SDO may be a trait that explains shared AV perceptions. This would help contextualize SAV acceptance research and align the emerging research with established social psychology.

SDO may be an important factor in acceptance of SAVs both because of the social situation and the novelty of the transport mode. Recent research has found that peoples’ inclination to support domination over groups of people, as measured by SDO, also extends to domination over the natural world (Milfont et al., 2018; Pratto et al., 1994). SDO has also been found to predict attitudes towards cyclists (Williams, 2022). Social change and innovation could also be linked to the conservative views associated with SDO (Becker, 2020). Therefore, those high in SDO may be less positive about SAVs because it is perceived as an innovative transport mode that also legitimizes action towards climate change. Simultaneously, SDO has long predicted inter-group attitudes and a desire to maintain hierarchy. If SAVs are seen as an arena for social equity and a place for random encounters, this may impact willingness to use such services for individuals higher in SDO. Discrimination between ridesharing passengers may discourage further use of such services, and SDO has previously been documented to drive such attitudes (Middleton and Zhao, 2019; Moody et al., 2019). SDO may thus represent an important and untested part of AV acceptance. Other personality traits may also play a role in determining whether people want to use SAVs.

### 2.3 Research question and hypotheses

The current study seeks to examine the acceptance of shared automated vehicles (SAVs). We investigate two main research questions: (1) how does changing information about the SAV service impact people’s perception about the service, and (2) how do different personality traits predict the general perception of the SAV service. Based on likely scenarios and the reviewed literature, we have developed three pairs of experimentally altered information given to participants. These are regarding seat configuration, autonomous capability, and ethnicity of co-passengers. We derive three exploratory hypotheses regarding these:

1 Does sitting in the direction of travel impact SAV perception?
2 Does having a steward on board impact SAV perception?
3 Does having differences in ethnicity in potential co-passengers impact SAV perception?

Furthermore, we hypothesize that the effects of our manipulations may be stronger for some personality types:

4 People higher in extraversion prefer social seating options.
5 People higher in agreeableness prefer stewardless autonomous driving.
6 People higher in neuroticism prefer having a human steward onboard.
7 People higher in SDO prefer homogenous co-passengers.

We will also explore the main effects of proposed personality and individual difference variables on the general perception of the SAV service.

## 3 Materials and methods

### 3.1 Sampling

Participants in this online survey were recruited in two ways. We found that SMS invitations only showed a response rate of 3%. We therefore invited participants from a previous survey who had agreed to be contacted again. This was originally a general sample of the Norwegian population, and we had consent to store their emails. This email list consisted of 8,892 unique addresses. The survey they had responded to was about infrastructure and maintenance for cyclists and pedestrians (parts of those results are published here: Aasvik and Bjørnskau, 2021). Eventually, we ended up with 2,141 respondents (approximately 20% response rate). The data collection period lasted from 9th of June 2022 until mid-September 2022.

Power analysis using the software G*Power suggested that we needed 960 respondents for this survey, 160 distributed across the six experimental conditions. This calculation used a medium effect size of *F2* = 0.15, an alpha level of 0.05 and a beta level of 0.80, and a set of 12 predictors. Most research using UTAUT-frameworks achieve larger effect sizes than this, suggesting that we may be more prone to type 1 errors than type 2 in this study. We planned on doing a version of test–retest reliability analysis by collecting twice as many respondents. This was rendered unnecessary because the two groups of approximately 1,000 respondents had close to identical scores on all included variables. Thus, we included all participants in the analyses. The data collection was vetted by Norwegian Agency for Shared Services in Education and Research (Sikt) and found to be in accordance with ethical and legal guidelines.

### 3.2 Experimental manipulation

All items in the survey were measured on a Likert-scale ranging from 1 “Totally disagree” to 5 “Totally agree” with a sixth option “Not relevant/do not know” unless otherwise specified. When calculating means of scales, we recoded “Not relevant/do not know” into the mid-point of the scale (3). To minimize participants’ need for conjecture about the largely unknown future SAV service, we included explanatory text and illustrations:

You will now receive information about a future bus service that may be common in Norway in a few years. The vehicles will look like small buses and be self-driving. You order and pay for the service through a smart-phone app. The bus will come and pick you up where you are or at a bus stop, and you may have to share it with others traveling in the same direction. This self-driving bus will only be available through order and will not necessarily follow usual bus stops.

**Figure 1:**
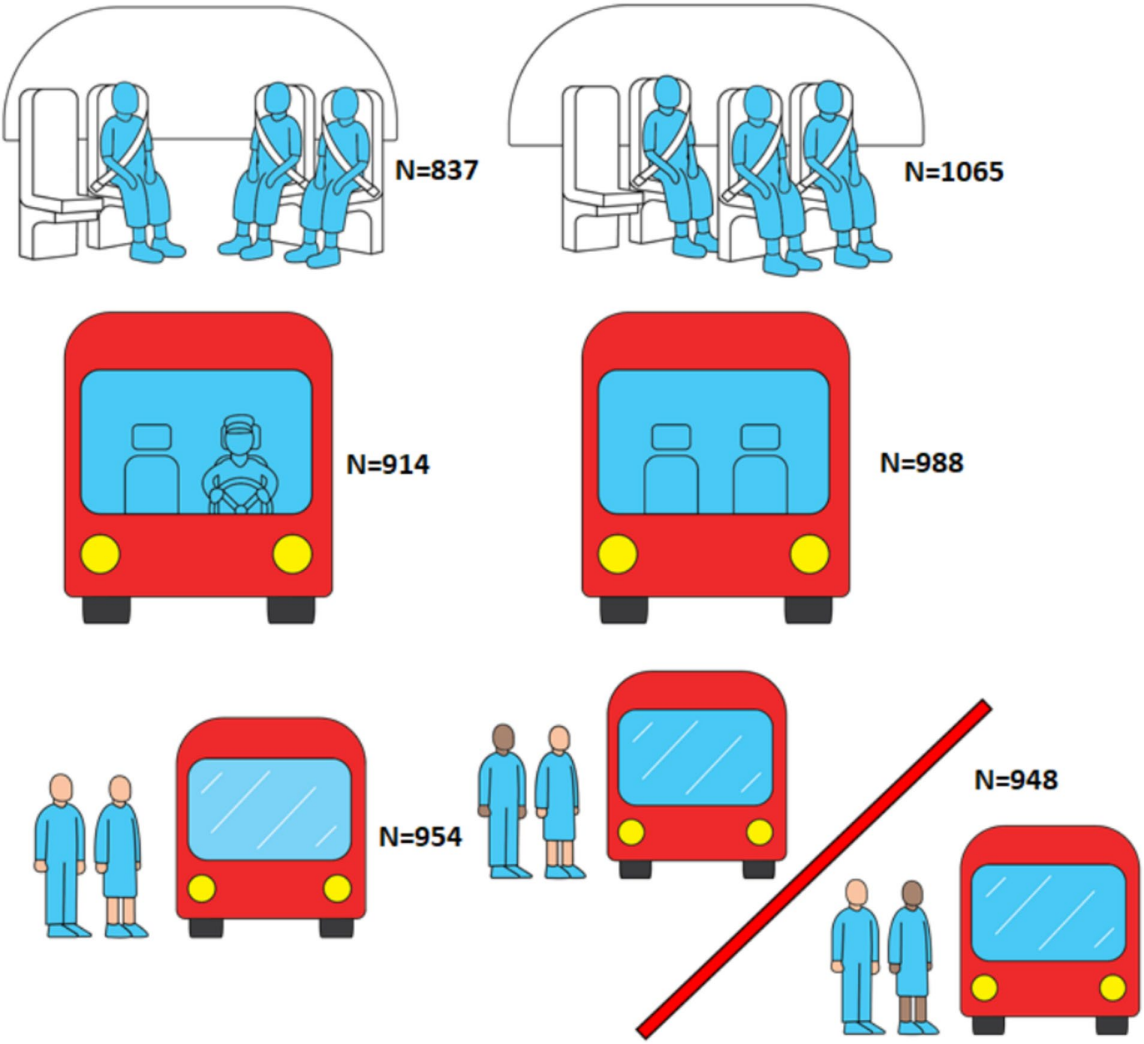
Illustrations given to participants about the future SAV service, with the number of people who saw each of the six conditions.

Thereafter followed three pairs of illustrations as shown in Figure 1. These were shown along with a short text explaining the illustrations’ content.

The two versions of the three pairs of illustrations were randomly shown to equal sub-groups of the sample. This means that there were eight groups (2 × 2 × 2) in total who saw different combinations of the information, and that each group worked as a control for the rest. For the mixed ethnicity condition, we had a set with a darker skinned woman and a darker skinned man to equalize any differences between the two. The information and illustrations were approved by the main public transport authority in the Oslo-region, Ruter to be representative of a realistic future scenario.

The survey first collected informed consent, basic sociodemographic information, and travel habits, as well as any AV experience. After this, we displayed the experimental information about SAVs. After the information, we presented MAVA-items before FFM and SDO. More sensitive topics, like SDO and questions about income and discrimination, were placed last in the survey.

### 3.3 General acceptance of shared automated vehicles

To create a concise measure of the MAVA, we looked at previous investigations of both MAVA and UTAUT. The current study uses the same data as a previous publication that investigated the structure of the MAVA-items as used in a SAV setting (Aasvik et al., 2024c). Here, analysis suggested that some items did not fit well with the conceptualization of a single acceptance factor. There was support for a single latent variable, the General Acceptance Factor (GAF). The current study takes this into account and uses the suggested 14-item aggregate measure of MAVA, henceforth referred to as the GAF. This 14-item GAF had a good Cronbach’s alfa at 0.92, suggesting good inter-item reliability (Lance et al., 2006). Furthermore, the GAF showed a strong linear relationship with intention to use, proving its’ utility as a precursor to behavioral intention. An elaboration on items can be found in the Appendix.

This measure of GAF has trust and utility as its’ most central components, but also encompasses safety, norms, hedonic motivation, climate action, and perceiving oneself to be in the target group for such a service. Items were measured on a Likert-scale ranging from 1 “Totally disagree” to 5 “Totally agree” with a sixth option “Not relevant/do not know”. When calculating the mean, we recoded 6 “Not relevant/do not know” into the mid-point of the scale (3). This is one of a few ways to deal with non-response options, and is found to create insignificant differences as compared to treating them as missing (Denman et al., 2018). This kind of recoding is justified as long as it makes sense as a neutral option in the context, which we believe it does for these questions where the mid-point probably is used interchangeably with the “not relevant/do not know” option (Nadler et al., 2015).

### 3.4 Five factor model

We relied on a version of the mini IPIP scales measuring the five factors in the Five Factor Model (FFM) using only four items per factor (Donnellan et al., 2006). This scale has previously been validated, translated, and used in similar contexts (Engvik and Føllesdal, 2005; Johansson and Fyhri, 2017). We calculated a mean score for each of the five factors. The score was coded so that a high score means that the respondent is higher on the trait label. Like the GAF, all items were measured on a Likert-scale ranging from 1 “Totally disagree” to 5 “Totally agree”. See Appendix for further details on wordings of items.

The scales showed varying degrees of internal reliability (Lance et al., 2006). Agreeableness’ Cronbach’s alfa was 0.61, conscientiousness had 0.51, extraversion had 0.84, neuroticism had 0.82, and openness had 0.67. These are mostly quite low. However, they are validated and supposed to cover a wide array of sub-traits.

### 3.5 Social dominance orientation

Social Dominance Orientation (SDO) is conceptualized to consist of two sub-scales: anti-egalitarianism and dominance. The short SDO scale uses only four items, but does not adequately cover both sub-scales (Pratto et al., 2013). We relied on this but opted for the shortest version that covers both scales, using six items in total. This scale has previously successfully been used in Norwegian (Johansson and Kunst, 2017; Milfont et al., 2018). The items were measured on a Likert-scale ranging from 1 “Totally disagree” to 5 “Totally agree”. The mean score was coded so that a higher score means more agreement with the scale items. The scale showed an acceptable Cronbach’s alfa of 0.79. See Appendix for further details on wordings of items.

### 3.6 Analysis

The analyses were performed in the Jamovi software (The Jamovi Project, 2021). We ended up with a large sample, suggesting that we need to consider the balance between type 1 and type 2 problems. By looking at effect sizes and not merely at *p*-values, we avoid some of the issues with potential false positives (Wasserstein and Lazar, 2016).

The current study shares data with another publication (Aasvik et al., 2024c). The anonymized dataset is published at the Open Science Framework (Available at: osf.io/4pgrj, Aasvik, 2022). They share the same participants, sociodemographic information, MAVA-items, and suggested extensions. However, while the current publication focuses on investigating the interaction between MAVA, experimental manipulations and individual level variables, the other publication focuses on investigating the component structure of acceptance. These study aims are different and involve using partly different sets of variables. They do inform each other but are developed as separate manuscripts.

We used ANOVA to test for main effects of our experiments. These tests were directly related to hypotheses one, two, and three. The next four hypotheses included interaction effects. These were tested in a multiple regression while controlling for main effects of the personality constructs and experiments. The regressions therefore also served to investigate main effects of personality traits.

#### 3.6.1 Data cleaning

Our gross sample consisted of 2,141 participants. Two of these were incomplete and one stated that their response should be removed due to dishonesty. An attention check was used, asking participants whether the seats they were shown were in the driving direction or facing the other passengers. Two hundred and thirty-six participants (11%) failed to pass. This left 1902 participants who were brought forward to analysis. This exceeds our previous estimate of 960 participants, meaning that our study is well-powered for its’ intended purposes.

## 4 Results

### 4.1 Descriptive results

Our sample consisted of 63% men. The most represented age categories were 50–59 and 60–69, with 5% younger than 30. Most of the sample (56%) reported having at least 3 years of university level education. This suggests an overrepresentation of both male, educated, and older participants. More than one third (37%) of the sample reported having seen a self-driving shuttle in traffic, and only 9% had never heard about pilots using self-driving shuttles. Table 1 presents means and standard deviations of the study constructs.

**Table 1:** Means and standard deviations of the study’s constructs, *N* = 1902.

Constructs	Mean	SD
Openness	3.28	0.67
Neuroticism	2.16	0.74
Conscientiousness	3.91	0.51
Agreeableness	3.85	0.51
Extraversion	3.06	0.76
SDO	1.83	0.67
General Acceptance Factor (GAF)	3.28	0.74

Constructs were measured on a five-point Likert scale.

Most scores are close to the mid-point of the Likert scale (3). People generally score low on neuroticism and SDO. The highest score is in conscientiousness. The mean GAF score is near the natural midpoint of the scale, indicating that the acceptance of SAVs, on average, can be described as moderate. There were no extreme skews or kurtosis among the variables.

### 4.2 Effect of experimental manipulations on general acceptance of SAVs

To test the effects of our experimental manipulation, we ran an ANOVA. We introduced the experimental conditions as independent factors predicting the GAF, as this is theorized to be a precursor to behavioral intention and behavior. This model, investigating the main effects of experimental manipulations, is presented in Table 2.

**Table 2:** Effects from the ANOVA investigating experimental manipulations’ effect on the GAF, *N* = 1902.

ANOVA predicting GAF	Yes	a	No	a	*F*	*p*	η^2^
	Marginal Means	(SD)	Marginal Means	(SD)			
Social seating	3.26	0.75	*3.29*	0.73	1.06	0.304	0.001
AV without driver	3.28	0.75	3.27	0.73	0.03	0.867	0.000
Mixed ethnicity	3.28	0.76	3.27	0.72	0.03	0.854	0.000

*p < 0.05, **p < 0.01, ***p < 0.001. ^a^ “Yes” and “No” refers to whether the label of the experimental condition was present or not.

The ANOVA results suggest that none of the experimental manipulations had a statistically significant main effect on respondents’ GAF scores. Additionally, the effect sizes (η^2^) are close to zero, suggesting that the manipulations explain virtually no variance in GAF. These findings imply that, in this context, participants’ general attitudes are not substantially influenced by seating arrangements, the presence of a driver, or the ethnic composition of the vehicle’s occupants. This may indicate that other factors, not captured by these manipulations, play a more critical role in shaping perceptions of SAVs.

### 4.3 Main and moderating effects of personality on GAF

Table 3 shows two separate multiple OLS regression models. In the first, we introduce personality constructs as predictors of GAF. In the second, we also include experimental effects and four interaction terms from our hypotheses.

**Table 3:** Predicting GAF scores based on personality, experimental conditions, and interaction effects.

	Stand. estimate	95% CI		*p*		Stand. estimate	95% CI		*p*	
SDO	−0.14	−0.19	−0.10	< 0.001***		−0.13	−0.19	−0.06	< 0.001***	
Extroversion	0.01	−0.03	0.06	0.535		0.01	−0.05	0.07	0.672	
Agreeableness	0.10	0.05	0.15	< 0.001***		0.08	0.01	0.15	0.024*	
Conscientiousness	−0.03	−0.08	0.01	0.161		−0.04	−0.08	0.01	0.154	
Neuroticism	−0.09	−0.13	−0.04	< 0.001***		−0.09	−0.15	−0.02	0.013*	
Openness	0.14	0.10	0.19	< 0.001***		0.14	0.10	0.18	< 0.001***	
Mixed ethnicity						0.00	−0.08	0.09	0.448	
Social seating						−0.06	−0.15	0.02	0.617	
AV without driver						0.02	−0.07	0.10	0.540	
SDO ✻ Mixed ethnicity						−0.03	−0.12	0.05	0.436	
Extroversion ✻ Social seating						0.01	−0.08	0.10	0.872	
Neuroticism ✻ AV without driver						0.00	−0.09	0.09	0.996	
Agreeableness ✻ AV without driver						0.04	−0.06	0.13	0.440	
Adjusted *R*^2^	*0.071*					*0.069*				

Multiple regression analyses (*N* = 1902). All variables z-transformed to alleviate issues with multicollinearity. *p < 0.05, **p < 0.01, ***p < 0.001.

The results demonstrate a link between some personality traits and SAV perception as measured by the GAF. SDO, openness, agreeableness, and neuroticism were significant predictors, with SDO and openness showing the largest standardized effects.

SDO and neuroticism are negatively related to the GAF, suggesting that higher levels of these are associated with less favorable SAV perceptions. Extroversion and conscientiousness did not significantly predict GAF levels, indicating that these personality traits may not play a substantial role in influencing this particular measure. Additionally, none of the experimental manipulations (mixed ethnicity, social seating, AV without a driver) nor their interactions with personality traits significantly predicted the dependent variable. This suggests rejection of all four study hypotheses. The adjusted R^2^ values indicate that much of the variance remains unexplained by the variables included in this model.

Furthermore, the impact of personality constructs is mostly unchanged between the two models. There is some change in their probability values, but the coefficients mostly remain the same. This suggests that both main and interactional effects of the experimentally altered information had an insignificant impact on SAV perception, nor did this information interact with different personality traits.

## 5 Discussion

The current study tested experimentally altering information about a realistic future SAV service. We tested the impact of this information on the general acceptance factor (GAF) suggested by previous research. Furthermore, we investigated how the GAF is impacted by different personality traits, and whether there is an interaction between information and personality traits. We fail to uncover any impact of our experimental conditions, neither main effects nor interactional effects. Four of six included personality traits significantly predict the GAF, with SDO, agreeableness, and openness showing the largest effects. This suggests that there are clear differences between personality traits in SAV perception. Effects from SDO and agreeableness highlight the importance of the social situation in ridesharing, as well as interpersonal trust and trust in automation. The lack of experimental effect is surprising and is discussed in the following sections.

### 5.1 Absence of experimental effects

Researchers have argued that the configuration showing more automation and more traditional driving-direction seating should be favored (Nordhoff et al., 2019a; Paddeu et al., 2020, 2021). This was not the case in the current study. Differences in perception of AVs implemented for public transport or private use have been found, i.e. comfort is more important in AVs employed for public transport (Bala et al., 2023). The same trends could have impacted our results causing a preference for riding in vehicle resembling a private vehicle with seats facing the direction of travel. However, no such effect was uncovered. Most pilots with SAVs have been using a steward who takes control of the vehicle if the SAV gets stuck (Hagenzieker et al., 2021; Pokorny et al., 2021). This lack of automation and sub-optimal performance of the vehicles may have had some adverse effects on people’s perceptions (Aasvik et al., 2024b). This may counter-act the previously noted effect where people grow more optimistic about the service the more they contemplate a SAV future (Tennant et al., 2016). Experiencing nascent technology may be sobering for people who grow optimistic about its’ future possibilities. Therefore, there may be conflicting and opposite forces impacting people’s perception of whether they are more in favor of having an on-board steward or not.

The experimental manipulation may have failed because the perceived differences between conditions were too small or unimportant. We excluded 11% of our gross sample after a manipulation check, suggesting that the remainder did perceive the details of the information shown. It is somewhat surprising that we failed to find effects of i.e. extraversion moderated by the orientation of the seats or neuroticism mediated by autonomous driving. Perhaps mere informational changes are too small to impact perceptions in this way as no further safety information was provided about the autonomous driving. People may expect the vehicle to be under supervision anyway, if not by an on-board steward, then by a remote operations center. Similarly, ridesharing may be considered an inherently social activity regardless of how the seating is arranged internally in the vehicle. While there are some disparate attitudes towards minorities in Norway (Brekke et al., 2020), the lack of effect of ethnicity on SAV perception may be explained by the country’s egality, the social desirability of the subject at hand, or the weakness of the manipulation in the current study (Hasell, 2023).

Having seating configurations that are backwards to the direction of travel have been found to negatively impact perceptions of AVs, and trust in particular (Nordhoff et al., 2019a; Paddeu et al., 2020). We should therefore expect this part of our experiment to negatively impact the GAF, as trust is one of its’ main constituents (Aasvik et al., 2024c). The experiment that first mentioned this effect used an on-site pilot of a shuttle with seating options in both directions. Our experiment was merely informational, but did not employ a within-subject test of seating direction. However, for our condition with seats facing each other, participants could still imagine themselves riding in the direction of travel. This could work to attenuate the effect of travel direction. Our manipulation focused on capturing the social aspect of this configuration, as well as the general evaluation. Because of this, we would argue that our null find does not suggest that there is no effect of seating configuration, but that our experiment was unable to capture its’ effect on trust. However, others have suggested that seating orientation does not impact factors such as trust and comfort (Paddeu et al., 2021). Other issues, like spreading disease or not feeling well when travelling backwards may also be related to seating configuration. The current study’s data collection was done shortly after the COVI-19 pandemic, which may have accentuated these issues for our analyses. Perhaps seating configuration in future vehicles should account for these additional issues, as well as people wanting to do other things than talk, like working or resting (Kwon and Ju, 2021).

Previous research has suggested that having a steward onboard may impede adoption of SAVs (Winter et al., 2019). No such effect was uncovered in our study, in line with other studies (Paddeu et al., 2021). This suggests that these effects may be quite small or pertain to specific situations, i.e. with higher speeds or to certain demographic groups, i.e. women (Aasvik, 2024). The current study did not mention operating speeds, but we can assume that people imagined that the service would comply with speed limits wherever it operates. The argument has been made for a while that bus and taxi drivers may become obsolete in the near future (Paul, 2017). Like elevator operators who became redundant at the advent of new automation, modern vehicles may simply need a push of a button to perform a task. However, the question remains how people will react to this abrupt change. There probably still exists some need for emergency connections for riders to feel safe, but the effect does not seem to be very articulated in the limited research investigating this. Perceptions of shared vehicles where there is no operator remain under-investigated.

Other information elements could be of further interest to manipulate. Research has found that intention to use automated shuttles is greater than intention to use robotaxis (Salladarré et al., 2021). Others state that automated shuttles are preferred over automated buses, ridesharing and robotaxis (Chng et al., 2022). This preference could translate into a preference for face-to-face-orientation of seats. We did not find such an effect here, either suggesting that something else drives the preference for shuttles. Further preferences are being driven all the way to their destination, having pick-up points closer to home, good availability, efficient and cheap services, and ways of mitigating socially unpleasant situations (Aasvik, 2024; Aasvik et al., 2024a). Rather than relying on these stated preference surveys, experimental research could add knowledge about how people perceive differences in realistic scenarios. Furthermore, key issues of trust, safety, and design features could be explored in this fashion. AVs seem to be subject to stricter safety evaluations than human drivers, partly because of an ‘algorithm aversion’ and illusory superiority among human drivers (Shariff et al., 2021). This is a phenomenon where people are more critically inclined towards automation and believe themselves to better drivers than the average. Such effects cooperate to impair the evaluation of SAVs. How information about safety and performance is presented could thus be key in people’s evaluation of such a service and could be investigated experimentally. We therefore still persist in the belief that such experiments still could be of great value, but that the experiments need to be stronger than mere information, and preferably in a real-life setting.

### 5.2 Personality traits and acceptance

None of the interactions between experiments and personality traits were significant, suggesting rejection of our four hypotheses regarding this. Four of our six included personality traits show significant impact on SAV perception as measured by the General Acceptance Factor (GAF). These findings suggest that there is room to seek standardization of AV and SAV acceptance research by connecting with established psychological frameworks of personality. Particularly for Social Dominance Orientation (SDO), which plays a significant role. This is what we would expect given the social hostility often displayed by those high in the trait (Kleppestø et al., 2020). It remains to be further examined whether this effect is interacting with the perceived social status of co-passengers, which has been shown to be influential in similar settings (Middleton and Zhao, 2019; Pratto et al., 1994). We did not find any interactions between mixed ethnicity in the illustrations and general perception of the service, but this manipulation may have been too weak or vague. Our findings do corroborate previous investigations that SDO impacts willingness to use ridesharing services (Moody et al., 2019), and that this may be due to discriminatory attitudes. The effect of SDO on the GAF may further suggest that people high in SDO are less inclined to use a novel transportation service because they do not encourage climate action and technological disruption. The opposition to social equity further seems to be a main driver for these individuals’ disinclination to use SAVs. Researchers have suggested that a focus on similarities between social groups, challenging traditional gender roles, and advocating for nurture over nature may decrease the effects of SDO (Dambrun et al., 2009; Ho et al., 2015; Wilson and Liu, 2003). This may be of importance for improving the overall acceptance of SAVs for people high in SDO.

Agreeableness, openness, and neuroticism all have significant effects in the regression model predicting the GAF. This suggests that these personality traits influence how people perceive different aspects of SAVs. Future research could therefore orientate towards integrating the Five Factor Model (FFM) of personality into their investigations of AV acceptance. Agreeable people may be more positive to the ridesharing experience, having more trust in technology and other people (Chien et al., 2016; Graziano et al., 1996). This main effect is supported in our research, but we do not find that the more social seating configuration further accentuates this effect. While previous research is somewhat divided on whether openness to experience translates to the field of SAVs, our results suggests that it does translate to being interested in trialing this novel technology (Barnett et al., 2015; Svendsen et al., 2013). Openness could also suggest that these respondents are more open to trying different modes of transport, rather than being locked into their current travel habits. Research has suggested a clear link between neuroticism and trust formation in AV technology (Kraus et al., 2021). Corroborating previous assumptions, respondents higher in neuroticism may perceive this novel service as less useful and have more negative feelings towards SAVs (Barnett et al., 2015; Charness et al., 2018; Svendsen et al., 2013). For this group, an extra focus on trust formation and safety interventions may be in order to increase their willingness to use SAVs. Having a test ride in a SAV service have been shown to improve perceptions, particularly for anxious riders (Feys et al., 2021).

Extraversion was hypothesized to be particularly effective in this context due to the social nature of SAVs (Charness et al., 2018; Qu et al., 2021), but we fail to find this in our model. This could be because the GAF-construct fails to adequately capture the essence of extraversion; namely the extent to which people prefer and enjoy social company. Thus foreclosing an effect on the GAF. Extraversion may still play a role in specific contexts, such as a larger vehicle or a setting with more implied people onboard. Trait conscientiousness has shown mixed impact on intention to use SAVs. Our results suggest no impact from the trait on the GAF. Conscientiousness have been found to predict increased concern about AV technology (Charness et al., 2018). This effect may be at work here, where it is more difficult for highly organized people to relinquish control to a SAV. Additionally, they may have concerns about the effectiveness of such a service, as ridesharing does include indirect routes to cater to all passengers’ needs. Similar concerns have been raised in similar contexts (Aasvik et al., 2024a).

Using an aggregate of our included MAVA-items to measure a general acceptance factor (GAF) necessarily masks some of the differences between MAVA sub-components. While previous research has suggested large enough inter-item correlation between MAVA-components to justify such a merged approach, it is important to keep the downsides in mind (Aasvik et al., 2024c; de Winter and Nordhoff, 2022). Neuroticism may play a role in safety evaluations and impacting trust, while conscientiousness may impact usefulness. However, these effects disappear when controlling for other personality factors in predicting an aggregated construct. There is a trade-off between parsimony and brevity in measurement on one side, and accuracy and explanatory power on the other side. Practical relevance also plays a role, where SAV operators may want to investigate some specific parts of a novel service. Future research should further investigate these nuanced differences.

### 5.3 Limitations

The data we employed in this study was mostly recruited from the general Norwegian population. This is not a representative sample, due to low response rates and difficulties in SMS distribution. Furthermore, most of the respondents were recruited from previously completed surveys about other transport-related subjects. Those who respond to surveys differ from the population at large, and especially those who do it twice (Groves and Peytcheva, 2008). We also had some overrepresentation of males, educated, and older participants in our sample. The study’s aims should not be largely impacted by this however, as we had no intention of extrapolating specific results from a smaller sample to the general population. Our aim was to build a better understanding the mechanisms of predicting SAV usage intentions, and this more general aim should be accomplishable with our sample.

Some of this study’s scales showed low internal reliability as measured by Cohen’s alpha. Agreeableness, openness, and conscientiousness had particularly low scores. Although the scales are validated and used successfully in similar contexts, it is important to note (Engvik and Føllesdal, 2005; Johansson and Fyhri, 2017). This could suggest that the items in the scale are poorly suited to be treated as representations of the underlying trait. Future research endeavors may take the opportunity to further seek a reliable measure of these three personality traits. However, the personality traits are theorized to represent different sub-components, and it is natural to expect some divergence of alpha values, especially for such short scales. While reversed items is a common way to mitigate straightlining in surveys, it may also introduce some noise and confusion (Van Sonderen et al., 2013). It is important to be mindful of reversing items as a potential double-edged sword, and particularly for such short scales. Although we tried to inform participants about a realistic future SAV scenario, the future of this service is still largely unknown. Our manipulation checks and exclusion criteria may have eliminated respondents systematically if they simply were less interested in seating configuration, and thus lower on SDO or extraversion. Future research should investigate whether and to what extent this is an issue, but we expect this issue to be minimal in our case. Respondents mostly do not have meaningful experience with the vehicle type in question, and this may bias the perception of them, as they are left to conjecture. As these vehicles become more readily available, researchers should strive for real life demonstrations and tests of actual services. We still believe that early efforts, like the one reported here, are an important foundation in the knowledge generation that is required for successful AV and SAV adoption in society.

## 6 Conclusion

In this study, we have investigated how experimentally altered information impacts perceptions of SAVs. We also employed key personality traits in predicting a General Acceptance Factor (GAF). We did not find that our informational experiment made any significant changes in people’s general acceptance. This could be because our manipulations were too weak to invoke substantial differences or because our chosen factors of seating configuration, on board steward, and mixed ethnicity were too unimportant to the GAF or SAV acceptability. We found that Social Dominance Orientation (SDO), agreeableness, openness, and neuroticism significantly impact the perception of SAVs. This suggests that some people are disinclined to use SAVs because of their cultural impact as technology disruption and the possible social equity to transport availability. Furthermore, some people more easily trust automation and are more open to changing their everyday travels. It remains an important finding that transport agencies should focus on building trust to the public when deploying SAV services. They should also look at ways of making the social situation within more predictable and satisfactory, for example by offering some information about co-passengers or creating travel communities of people with similar travel habits (Israel and Plaut, 2024). Research using real life vehicles in operation could build on these results and test how to make SAVs desirable for the wider public.

## Data Availability

The datasets presented in this study can be found in online repositories. This data can be found here: https://doi.org/10.17605/OSF.IO/4PGRJ.

## Footnotes

^1^We refer to automated vehicles as partially autonomous vehicles. These systems are not fully capable of self-driving. We refer to autonomous vehicles as the final development of the technology; a fully self-driving vehicle at SAE-level 5 (SAE International, 2021).
