# Supplementary material for: Exploring the general acceptance factor for shared automated vehicles: the impact of personality traits and experimentally altered information
Source: Front Psychol. 2025 Apr 9;16:1531386. doi: 10.3389/fpsyg.2025.1531386 (PMC12014631; doi:10.3389/fpsyg.2025.1531386)
Supplement: Supplementary file 1 [file Supplementary_file_1.docx]

**1 Appendix**

**1.1 Survey design elaboration**

**1.1.1 MAVA**

In this study, we wanted to test an aggregate MAVA-construct in an experimental setting, and whether this construct is predicted by personality traits. Much research has been conducted with the original UTAUT framework, and this is similar (Venkatesh et al., 2016). Recently, the UTAUT was again adapted to acceptance of automated vehicles (Bellet & Banet, 2023). Our conceptualization is based on these, but also adapted to our context of SAVs. The current study shares data with a previous publication (Aasvik, Ulleberg, et al., 2024). This publication examined the scale and component structure of UTAUT/MAVA-items adapted to SAVs. They found evidence for the redundancy inherent in the model, suggesting that the items represent a latent General Acceptance Factor (GAF). Furthermore, this GAF could be represented by as few as two variables. This examination also found grounds for inclusion of some extraneous variables and exclusion of others. For the purposes of the current study, we will use the following conceptualization using 14 items. Table 4 presents these items.

*Table 4. MAVA-factor, wording, translations, and most important sources for MAVA-items.*

| MAVA-factor | Norwegian | English | Most relevant sources |
| --- | --- | --- | --- |
| **Easy to use** Effort expectancy/Facilitating conditions | Jeg tror det ville vært enkelt for meg å bruke denne busstjenesten | I believe it would be easy for me to use this bus service | (Acheampong & Cugurullo, 2019; Korkmaz et al., 2021; Sener et al., 2019; Venkatesh et al., 2012) |
| **Safe wait while dark** Safety/Perceived risk/Trust | Jeg ville følt meg trygg mens jeg ventet på en slik buss når det er mørkt | I would feel safe while waiting for such a bus when it's dark | (Acheampong & Cugurullo, 2019; Backer-Grøndahl et al., 2007; Bansal et al., 2016; Korkmaz et al., 2021; Nordhoff, Kyriakidis, et al., 2019; Sener et al., 2019) |
| **Afraid of hacking (R)** Safety/Perceived risk/Trust | Jeg ville vært redd for at noen kunne hacket datasystemet til bussen | I would be afraid that someone could hack the bus's data system | (Acheampong & Cugurullo, 2019; Backer-Grøndahl et al., 2007; Bansal et al., 2016; Korkmaz et al., 2021; Nordhoff, Kyriakidis, et al., 2019; Sener et al., 2019) |
| **Risky use (R)** Safety/Perceived risk/Trust | Denne bussen ville være risikabel å ta i bruk | This bus would be risky to use | (Acheampong & Cugurullo, 2019; Backer-Grøndahl et al., 2007; Bansal et al., 2016; Korkmaz et al., 2021; Nordhoff, Kyriakidis, et al., 2019; Sener et al., 2019) |
| **Improve traffic safety** Safety/Perceived risk/Trust | En sånn buss ville økt trafikksikkerheten | Such a bus would increase traffic safety | (Acheampong & Cugurullo, 2019; Korkmaz et al., 2021; Nordhoff, Kyriakidis, et al., 2019; Raue et al., 2019; Xu et al., 2018) |
| **Trust the bus** Safety/Perceived risk/Trust | Jeg ville stolt på en sånn buss | I would trust such a bus | (Choi & Ji, 2015; Lee & See, 2004; Nordhoff, Kyriakidis, et al., 2019) |
| **Useful to me** Service and vehicle characteristics/perceived benefits/performance expectancy | Jeg tror denne typen buss vil være nyttig for meg | I think this type of bus will be useful for me | (Acheampong & Cugurullo, 2019; Korkmaz et al., 2021; Nordhoff, Kyriakidis, et al., 2019; Raue et al., 2019; Xu et al., 2018) |
| **Better than regular bus** Service and vehicle characteristics/perceived benefits/performance expectancy | En sånn buss ville vært bedre enn en tradisjonell buss | Such a bus would be better than a traditional bus | (Korkmaz et al., 2021; Sener et al., 2019; Venkatesh et al., 2012) |
| **Injunctive norm**  Social influence/Norm | Jeg tror andre syns det er bra at jeg bruker en sånn buss | I think others would think it's good that I use such a bus | (Acheampong & Cugurullo, 2019; Ajzen, 1991; Korkmaz et al., 2021; Sener et al., 2019; Venkatesh et al., 2012) |
| **Descriptive norm** Social influence/Norm | Jeg tror folk flest vil ønske å bruke en sånn buss | I think most people would want to use such a bus | (Acheampong & Cugurullo, 2019; Ajzen, 1991; Korkmaz et al., 2021; Sener et al., 2019; Venkatesh et al., 2012) |
| **Would be entertaining** Hedonic motivation | Å bruke en slik buss ville vært underholdende | Using such a bus would be entertaining | (Hohenberger et al., 2016; Kyriakidis et al., 2015; Sener et al., 2019) |
| **Climate concerns** Extraneous variable | Det er viktig å gjøre tiltak innen transport for å nå klimamålene | It is important to take action in transportation to achieve the climate goals | (Aasvik, Ulleberg, et al., 2024) |
| **Indifference towards bus** Extraneous variable | Jeg er likegyldig til dette tilbudet med selvkjørende buss | I am indifferent to this service using a self-driving bus | (Aasvik, Ulleberg, et al., 2024) |
| **In target group** Extraneous variable | Tenker du at du er del av målgruppen for sånne busser? | Do you think you are part of the target audience for such buses? | (Aasvik, Ulleberg, et al., 2024) |

MAVA-items were constructed to keep the scale as short as possible, while covering all aspects of the framework, and covering factors that are particularly relevant to the SAV context in Norway. This resulted in some factors being measured by several items. Items were also adapted to the fact that most people are unaware of the future capabilities of such a service. This also meant that we only prioritized including one item measuring service and vehicle characteristics (“Better than traditional bus”), and this was included in the aggregate ‘Performance expectancy’.

**1.1.2 FFM**

Short forms of FFM have been developed and tested previously with as few as four items for each factor (Donnellan et al., 2006). These have been translated to Norwegian and used in similar contexts (Engvik & Føllesdal, 2005; Johansson & Fyhri, 2017). We used these versions for our study, which are presented in table 5.

*Table 5. FFM-items and their translations.*

| FFM-factor | Norwegian | English |
| --- | --- | --- |
| E | Er pratsom | Am talkative |
| E (R) | Har en tendens til å være stille av meg | Tend to be quiet |
| E (R) | Kan være sky og reservert | Can be shy and reserved |
| E | Er utadvendt og sosial | Am outgoing and social |
| A | Er hjelpsom og uegoistisk ovenfor andre | Am helpful and selfless towards others |
| A (R) | Kan være kald og fjern | Can be cold and distant |
| A | Er hensynsfull og vennlig overfor de fleste mennesker | Am considerate and friendly towards most people |
| A (R) | Kan noen ganger være uhøflig mot andre | Can sometimes be rude to others |
| C | Gjør en grundig jobb | Do a thorough job |
| C (R) | Kan være uforsiktig | Can be careless |
| C (R) | Har en tendens til å ha lite orden på tilværelsen | Tend to have little order in life |
| C | Legger planer og følger dem opp | Make plans and follow them through |
| N | Er deprimert, nedtrykt | Feel depressed and blue |
| N (R) | Er avslappet, takler stress godt | Am relaxed, handles stress well |
| N | Bekymrer meg mye | Worry a lot |
| N | Blir lett nervøs | Get easily nervous |
| O | Er original, kommer med nye ideer | Am original, come up with new ideas |
| O | Har livlig fantasi | Have a lively imagination |
| O | Liker å spekulere, leker med ideer | Like to speculate, play with ideas |
| O (R) | Har få kunstneriske interesser | Have few artistic interests. |

FFM factors using only four items may show lower alpha levels. However, they are previously validated to represent the larger latent factors to a satisfying extent. The short form of the scales also means that alpha rarely is suggested to improve by deleting items.

**1.1.3 SDO**

To measure SDO properly one should include both sub-factors of the scale, Anti-Egalitarianism and Dominance. This was done using six items validated and translated in previous research (Ho et al., 2015; Johansson & Kunst, 2017; Kleppestø et al., 2020; Milfont et al., 2018; Pratto et al., 2013). Additionally, we reversed the scale for half the sample, meaning that the Likert anchors for group A read: 1 “Totally disagree” to 5 “Totally agree” and for group B they read: 1 “Totally agree” to 5 “Totally disagree”. This created a small difference between the two means (*F* (df=1, 1839) = 4.75, *p* = 0.029). The mean for group A was 1.8 and 1.9 for group B. For all other later analyses, these are merged in the direction of group A. Table 6 shows the items used.

*Table 6. Wording and translations for SDO-items.*

|  | Norwegian | English | SDO-category |
| --- | --- | --- | --- |
| 1 | Når vi skal prioritere, må vi ta hensyn til alle grupper (R) | **In setting priorities, we must consider all groups** | AE |
| 2 | Vi bør ikke drive frem likhet mellom grupper | **We should not push for group equality** | AE |
| 3 | Likhet mellom grupper bør være vårt ideal (R) | **Group equality should be our ideal** | AE |
| 4 | Overlegne grupper bør dominere underlegne grupper | **Superior groups should dominate inferior groups** | D |
| 5 | Et ideelt samfunn trenger at noen grupper er på topp og andre på bunn | An ideal society requires some groups to be on top and others to be on the bottom | D |
| 6 | Det er urettferdig å jobbe for at grupper skal være likeverdige | It is unjust to try to make groups equal. | AE |
